# A Kikuchi-Fujimoto Disease Case Mimicking T Cell Lymphoma with Prolonged Fever

**DOI:** 10.1155/2014/957134

**Published:** 2014-12-09

**Authors:** Serap Yadigar, Ilker Inanc Balkan, Nese Saltoglu

**Affiliations:** ^1^Department of Internal Medicine, Cerrahpasa Medical Faculty, Istanbul University, 34098 Istanbul, Turkey; ^2^Department of Infectious Diseases, Cerrahpasa Medical Faculty, Istanbul University, 34098 Istanbul, Turkey

## Abstract

Kikuchi-Fujimoto disease (KFD) is a self-limited disease characterized by necrotizing lymphadenitis. Although cervical lymphadenitis in young women is the most familiar clinical presentation, it may take place in the etiology of fever in cases presenting with fever of unknown origin. A 33-year-old male case admitted with fever, nausea, vomiting, weight loss, and leukopenia for one month, subsequently developing axillary lymphadenopathy during followup, diagnosed as KFD with typical histopathological findings, and showing full recovery after the excision of lymph node was presented in this report.

## 1. Introduction

Kikuchi-Fujimoto disease (KFD) is a self-limited disease characterized by necrotizing lymphadenitis [1]. The disease has firstly been defined in 1972 in Japan and is most commonly seen in Far-Eastern Asian countries [2]. Because of the rarity of the disease in Mediterranean communities, it might be overlooked in the differential diagnosis. The majority of the cases are composed of females under the age of 30. The disease is most commonly presented with cervical lymphadenopathy, accompanied with fever and findings of upper respiratory tract infection in some cases. Night sweats might be present while weight loss is rare. Leukopenia, impaired liver functions, and increased erythrocyte sedimentation rate are common [3]. Giant histiocytes with focal reticular cell proliferation and marked nuclear residues are noticeable in histopathology [4–6]. An unusual case of KFD admitted with fever of unknown origin and mimicking T cell lymphoma is presented in this report.

## 2. Case Report

**Figure 1:**
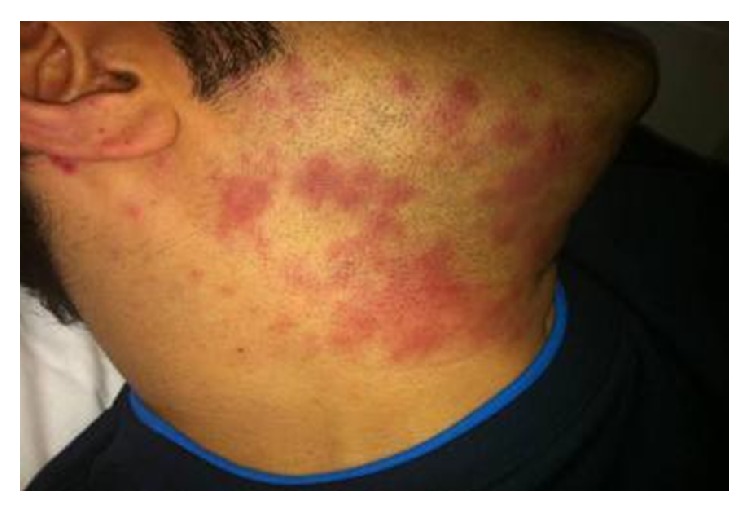


A 33-year-old otherwise healthy male was hospitalized in our clinic due to fever continuing for a month and a marked rash on his face and neck emerging within the last week. His fever was rising 4-5 times a day with chills and reaching up to 39°C. He had a newly appearing nonpruritic rash, noticeable on his face and neck with a pink-purple color and a diameter of 2-3 cm (Figure 1). He complained of nausea, vomiting, weakness, and weight loss of about 20 kg for the last 3-month period.

His physical examination revealed fever of 38,5°C with normal heart rate (88/rpm) and arterial blood pressure (110/70 mmHg). There was no peripheric lymphadenopathy (LAP) or organomegaly on admission. Examinations of other systems were also normal. No symptom or sign of any rheumatological disease was detected in detailed history, including SLE and vasculitis.

Laboratory results were as follows: WBC: 850/*μ*L, neutrophils: 320/*μ*L, lymphocytes: 430/*μ*L, hemoglobin: 12,3 g/dL, hematocrit: 39%, and PLT: 155 000/*μ*L. Erythrocyte sedimentation rate was 48 mm/h, CRP was 23 mg/L (nR: 0–5 mg/L), aspartate amino transferase (AST) was 58 U/L, alanine amino transferase (ALT) was 38 U/L, blood urine nitrogen (BUN) was 14 mg/dL, and serum creatinine (SCr) was 0.6 mg/dL. Anti-dsDNA, anti-RO, anti-LA, P-ANCA, and C-ANCA were negative while ANA was slightly positive. Serological tests for EBV, HIV, parvovirus B19, rubella, measles, mumps, RSV, HHV 6, HHV 8, parainfluenza virus, and* Toxoplasma gondii* were negative. Multiple mediastinal lymph nodes in 1 cm diameter were detected in thorax CT.

Because the patient had high fever, his general condition was moderately deteriorated and his neutropenia was deepening (170/*μ*L); meropenem 1 gr q8h was initiated empirically. However he did not respond and it was discontinued on day 8. PET-CT was performed to search for the etiology of fever. Multiple hypermetabolic (SUV_max⁡_ = 9,7) lymph nodes located supra- and infradiaphragmatically, those with suspicion of malignancy (significantly in right axillary and mediastinum), were detected. In addition to the ongoing fever and neutropenia, newly emerging axillary and cervical lymph nodes were detected during daily visits and the patient was referred to the haematology department for consultation. The microscopic and histopathological examinations of the bone marrow were normal. Excisional biopsy of the right axillary lymph node was performed.

The histopathology of the lymph node yielded nonsuppurative, coagulative foci of fibrinoid necrosis surrounded by histiocytes and immunoblasts, comprising diffuse karyorrhectic debris, particularly in the paracortical area. Plasmacytic infiltration areas were noticed. CD8 positive T cell infiltration was observed in the immunohistochemical staining while no accumulation was detected in PAS staining. Acid fast staining did not yield any organism. These findings were compatible with KFD.

A rapid recovery was observed after excision of the axillary lymph node and no specific treatment other than low dose NSAIDs was required. The rash was resolved spontaneously during followup. The patient was discharged with recovery on the 17th day of hospitalization and remained well in the regular outpatient follow-up visits for 6 months. Complete regression of lymph nodes was observed in the control chest CT at the 6th month follow-up visit.

## 3. Discussion

The patient was admitted with fever of undefined origin (FUO), neutropenia, rash, and subsequently emerging lymphadenopathies in the followup. Because fever, rash, neutropenia and slightly elevated levels of CRP (4-5-fold), ESR, and liver enzymes are common features of viral infections, viral serology was initially searched and found negative.

Sweet's syndrome may associate malignancies and present with fever, rash, and neutropenia. It was excluded with skin biopsy in our case. The hypermetabolic lymph nodes detected in PET-CT suggested T cell angioimmunoblastic lymphoma but it was ruled out with the histopathology of bone marrow and axillary lymph node. Histopathology of axillary lymph node was typical for KFD.

KFD is a rare disease that should be considered in the differential diagnosis of patients admitted with cervical lymphadenopathy and FUO. Although cervical lymphadenopathy is almost an absolute rule with very high frequency (100%), axillary and mesenteric lymphadenopathy is rare and only one-third (35%) of KFD cases present with fever [5]. The signs of the disease usually become evident in 2-3 weeks. Unlike the literature, the time interval between initial symptoms and the diagnosis was more than 3 months in our case.

Rash (10%), arthralgia (7%), arthritis (7%), weight loss (5%), night sweats (3%), hepatomegaly (3%), splenomegaly (2%), mild to moderate increase in erythrocyte sedimentation rate, and liver transaminases are also reported in KFD case series [7, 8]. Leukopenia is reported in 20–32% of the KFD patients. Thrombocytopenia and pancytopenia are less frequently reported. Anemia of chronic diseases would develop in severe cases. Similar to the literature, our patient had night sweats, arthralgia, and constitutional symptoms. Prominent nausea and vomiting at the onset led to significant (20 kg) weight loss in the first 2 months and suggested a gastrointestinal disease or malignancy. Pathological lymph nodes, those that were not remarkable in the initial physical examination, became palpable axillary after 1 week of hospitalization and also were detected with PET-CT as they scattered in different locations.

KFD is thought to develop on the basis of autoimmunity; however, the exact etiology still remains unknown [4–6]. Autoimmune response to various pathogens including EBV, HHV 6, HHV 8, HIV, parvovirus B19, Paramyxoviridae, parainfluenza virus,* Yersinia enterocolitica*, and* Toxoplasma gondii* is probably responsible for the disease [9–12]. The relation between KFD and SLE is not thoroughly understood and the histopathological findings are similar. SLE was excluded with negative clinical and serological markers in our case [7, 13]. Any related viral or autoimmune diseases were not detected. The definite diagnosis of KFD was established by histopathological study, as reported in the literature [14].

KFD is included in the differential diagnoses of malignant lymphomas, particularly angioimmunoblastic T cell lymphoma which may mimic rheumatic diseases with arthralgia, rash, and ANA positivity (14, 15). It is emphasized in a review of 244 KFD cases published by Kucukardali et al. in 2007 that lymphomas should be excluded in differential diagnosis [7]. The comparison of these 244 cases and our case is shown in Table 1.

KFD is a benign disease with no known specific treatment that resolves spontaneously within 1 to 4 months. Recurrence is rarely reported while high incidence of relapse is common in cases with ANA positivity [15]. The case presented here received no specific treatment other than short term NSAIDs and rapidly recovered after the excision of axillary LAP with no relapse or recurrence during the six-month followup after discharge.

As a benign disease, KFD should not be overlooked in the differential diagnosis of a patient with these symptoms and signs which may mislead the clinician to a certain type of non-Hodgkin's lymphoma, where the picture is not so promising.

## Figures and Tables

**Table 1:** Laboratory findings of KFD cases.

	Reported case series (7) (*n* = 244)	Present case
Leukopenia	43 (18%)	Positive
Leukocytosis	5 (2%)	Negative
Anemia	23 (9%)	Positive
Thrombocytopenia	10 (4%)	Negative
Elevated ESR	40 (16%)	Positive
Elevation in AST and ALT	19 (8%)	Positive
Elevated LDH	15 (6%)	Positive
Positivity of viral serology	13 (5.3%)	Negative
ANA positivity	18 (7%)	Slightly positive
Other serological abnormalities	8 (3%)	Positive (Rose Bengal false positivity)
